# Supplementary material for: Conditional chemoconnectomics (cCCTomics) as a strategy for efficient and conditional targeting of chemical transmission
Source: eLife. 2024 Apr 30;12:RP91927. doi: 10.7554/eLife.91927 (PMC11060718; doi:10.7554/eLife.91927)
Supplement: Figure 3—source data 3. [file elife-91927-fig3-data3.zip › Figure 3 source data3 part 1.zip!/Figure 3-Source data 3-part1/1071f2-1-CRISPResso_Report_ztwktvfp.zip!/CRISPResso_on_1071f2_4/4d.Position_dependent_average_indel_size.pdf]

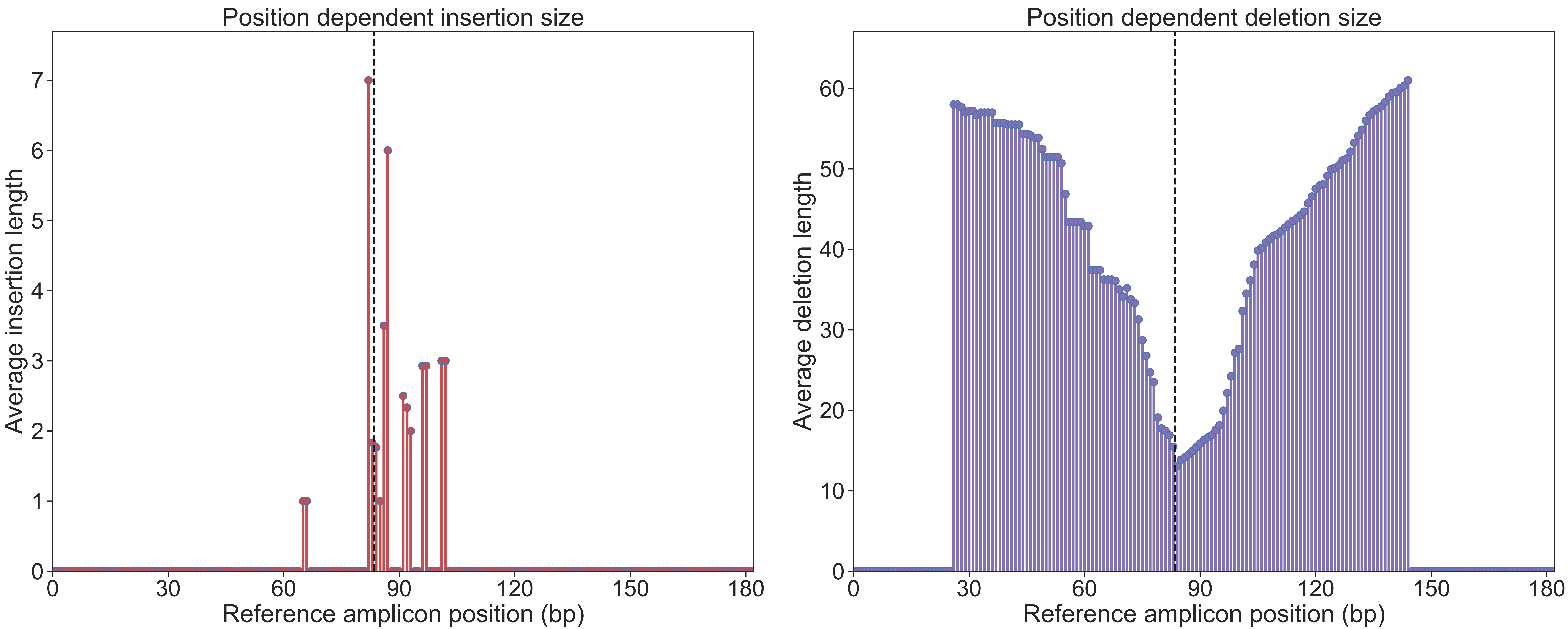
Position dependent insertion size
Average insertion length
Reference amplicon position (bp)
0
1
2
3
4
5
6
7
0
30
60
90
120
150
180
Position dependent deletion size
Average deletion length
Reference amplicon position (bp)
0
10
20
30
40
50
60
0
30
60
90
120
150
180
